# Could titanium oxide coating from a sol–gel process make stone baskets more resistant to laser radiation at 2.1 μm?

**DOI:** 10.1186/1477-5751-11-15

**Published:** 2012-10-19

**Authors:** Jens Cordes, Felix Nguyen, Frank Heidenau, Dieter Jocham

**Affiliations:** 1 Clinic of Urology, University of Lübeck, University Medical Center Schleswig-Holstein, RatzeburgerAllee 160, D-23538, Lübeck, Germany; 2 BioCerEntwicklungs GmbH Bayreuth (BioCer development Bayreuth), Bayreuth, Germany

## Abstract

**Background:**

Stone baskets could be easily destroyed by Holmium:YAG-laser at an endourologic treatment, with respect to this, we try to improve the resistance by coating them with a titanium oxide layer. The layer was established by a sol–gel-process.

**Materials and methods:**

Six new baskets (Equadus, Opi Med, Ettlingen, Germany) were used: 1.8 Ch. with 4 wires (diameter 0.127 mm). Three baskets were coated with a layer of titanium oxide established by a sol–gel process at the *BioCerEntwicklungs GmbH* in Bayreuth (~100 nanometres thickness). The lithotripter was a Holmium:YAG laser (Auriga XL, Starmedtec, Starnberg, Germany). 10 uncoated and 10 coated wires were tested with 610 mJ (the minimal clinical setting) and 2 uncoated and 2 coated wires were tested with 110 mJ. The wires were locked in a special holding instrument under water and the laser incident angle was 90°. The endpoint was gross visible damage to the wire and loss of electric conduction.

**Results:**

Only two coated wires resisted two pulses (one in the 610 mJ and one in the 110 mJ setting). All other wires were destroyed after one pulse.

**Conclusion:**

This was the first attempt at making stone baskets more resistant to a Holmium:YAG laser beam. Titanium oxide deposited by a sol–gel-process on a titanium-nickel alloy did not result in better resistance to laser injuries

## Introduction

An increase in the prevalence of urolithiasis to 5%, i.e. an increase of 25 percent within 20 years, was ascertained in Germany in 2000 [1].

The further development of instrumental technique towards extremely thin and even flexible ureteroscopes as well as modern lithotripsy procedures with various energy sources has once again placed special emphasis on endoscopic and percutaneous minimally invasive techniques [2].

One endoscopic procedure is endourologic lithotripsy in which the stone is destroyed in the ureter. Sometimes the stone is simultaneously stabilized by a stone basket. These baskets are made of nitinol a shape memory alloy of nickel and titanium (melting point ~ 1300°C) [3]. This stabilization of the stone in the basket could be on purpose or by accident. By accident means that an impaction in the ureter occurs by an extraction of a stone with a basket. Stabilization on purpose means to avoid a retropulsion into the kidney during a lithotripsy with a stone in the stone basket. At this part of the operation stone baskets have been frequently destroyed [4]. This severing of wires can lead to ureteral trauma due to hook formation [5]. On the other hand it could release the impacted stone in the basket from the basket by destructing all wires of the basket [6].

How quickly fragmentation with the laser occurs has been examined in vitro by Honeck et al. [7]. Baskets with a diameter of 3 Ch. were destroyed in 15–34 seconds and tipless Nitinol baskets (1.8 Ch. diameter) were destroyed in 1–4 seconds with a pulse energy of 0.8 and 2 J and a pulse frequency of 5 Hz. The guidance of the optical fibre occurred by means of a cystoscope in a container filled with water.

Cordes et al. [8] investigated four lithotripter and four different types of stone baskets. In this study they showed that the resistance of the baskets depends on the thickness of the wires. Also plaited wires seem to be more resistant to the radiation of the laser. An overview of accidental fragmentation of dormia basket and guidewire showed that this problem should be further investigated [9].

This study attempted to make stone baskets more resistant to a Holmium:YAG-laser by coating them with a titanium oxide layer established by a sol–gel process. This process was investigated by *BioCer,* and is normally used for polymer medical implants such as hernia meshes or hard-tissue implants. Because of the biocompatibility, the industrial availability and clinical experience with this layer we tested this layer on the basket-wires. The crystal class is tetragonal (rutil) which is the most common form of Titanium Dioxide. Rutil is hard, chemically resistant and has a high refractive index.

## Material and methods

**Figure 1:**
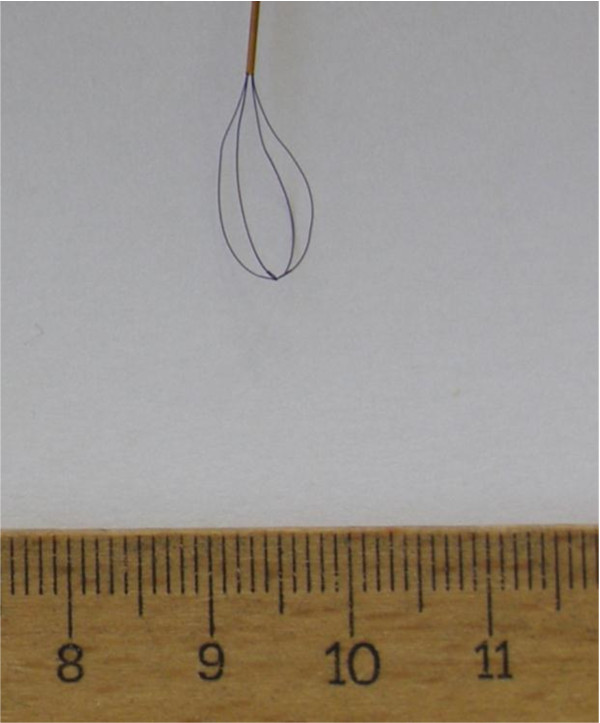
Stone basket with 4 wires 1,8 F(Equadus Opti Med, Ettlingen, Germany).

The lithotripter was a Holmium:YAG laser (Auriga XL, Starmedtec, Starnberg, Germany). Six new baskets (Equadus Opti Med, Ettlingen, Germany), tipless basket, 4 wires (diameter 0.07 mm), 1.8 F, nitinol) were used (Figure 1).

**Figure 2:**
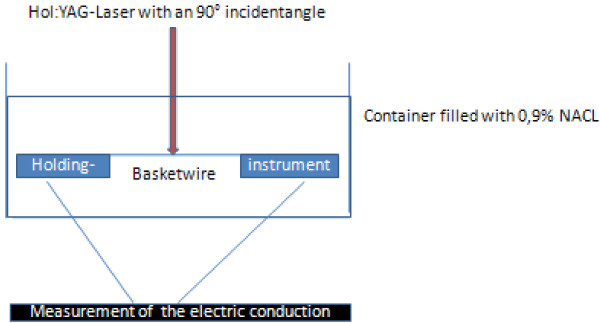
Schematic of the experimental setup.

Three baskets were coated with a layer of titanium oxide established by a sol–gel process at the *BioCerEntwicklungs GmbH* in Bayreuth (~100 nanometres thick). At this process only the surface of the basket wire is changed. The retention of this new surface was extremely close. In a study with mash grafts for hernioplasty it could not be broken off by mechanical force [10]. The wires were locked in a special holding instrument under water and the laser incident angle was 90° (Figure 2).

10 uncoated and 10 coated wires were tested with 610 mJ the minimal clinical setting. After seeing no significant difference in the destruction time the energy for 2 uncoated and 2 coated wires was reduced to 110 mJ.

**Figure 3:**
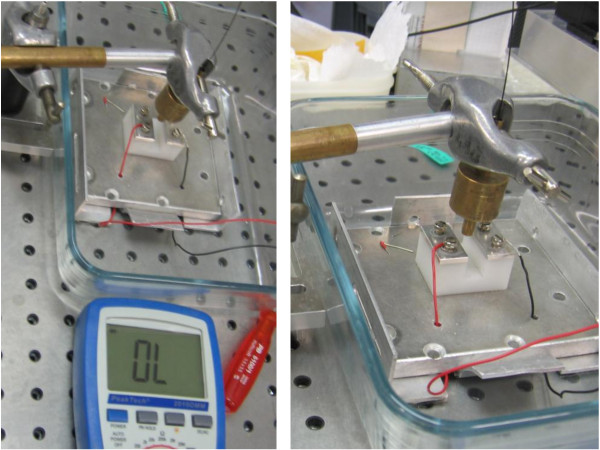
The investigational setting with the “multimeter”, the holding instrument and the laser fibre with a 90° incident angle.

The time and the pulses until destruction were measured and were documented by video. The endpoint was gross visible damage to the wire and loss of electric conduction. It was measured by a Multimeter (2010 DMM, Peaktech, Ahrensburg, Germany). The Modus called “Durchgangsprüfung mit Summer” in which a permanent sound signal indicates an intact conduction and a loss of this signal shows an interruption of conduction (Figure 3).

The laser was set to a repetition rate of 8 Hz and pulse energy of ~ 610 mJ. The laser fibre diameter was 365 μm and was in direct contact with the wire. The wavelength was 2.1 μm and the pulse period was 100-300 μs.

## Results

**Figure 4:**
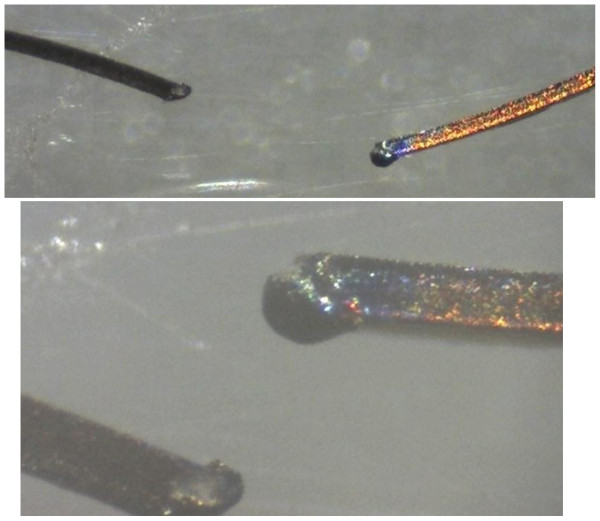
Microscopic picture of the gross visible damage of the wire with the typical melting drop (upper picture with a magnification of 60 and lower picture with a magnification of 200).

At the 610 mJ setting one coated wire was destroyed after 2 Pulses. All other wires were destroyed after 1 Pulse (9 coated and 10 uncoated wires) When the energy was reduced to 110 mJ one coated wire needed 2 Pulses. The others were destroyed after one Pulse (1 coated and 2 uncoated wires). All wires had a gross visible damage and the sound signal of the multimeter for an intact conduction stopped (Figure 4).

## Discussion

Further, prior work showed how baskets fracture across various compositions and configurations within modest range of Ho:YAG power settings [11]. This was a first attempt to improve the resistance of stone baskets to a laser beam of a Holmium:YAG laser. Nitinol is a self-passivating material. It naturally forms a surface oxide layer mainly TIO2-based with minimal amounts of nickel that protects the base material from general corrosion [12]. This layer is only some atom-layers thin and mechanically unstable [13]. By comparison with vapour deposition methods such as ion beam enhanced deposition, arc ion plating etc., by which it is difficult to form uniform coatings on the substrates with complex shapes, the sol–gel coating technique has advantages with regard to the independence of substrate shape (stone baskets) and adequate control of coating composition, thickness and topography [14]. This process is normally used in multiple layers for making glass harder [15] or for medical applications such as a functional coating for hard tissue implants [16]. In the present case, the authors assumed that the layer would also raise the resistance of the surface. Titanium oxide layers have optical effects and could raise reflection [15]. In the setting by Cordes et al. 2011 [8] it took some seconds to destroy the wires of the small basket. This differs from the present results and can be attributed to the clinical setting, in which Cordes et al. [8] used a renoscope, an artificial ureter and a basket that contained a stone. It appears that in this setup the wire was not fixed as closely as in the holding instrument in the current study, and that the laser incident angel is also important for the resistance. This is also shown in the experimental study of Freiha et al. in which a guidewire-damage varied with the inverse of the cosine of the incident angle [17].

Over all only two coated wires resisted 2 Pulses instead of one. It must be stated that the power of the laser for such a thin wire is too great for any significant differences to be observed. Maybe we should take a thicker basket and multiple layers of titan dioxide.

We think further investigations of wire materials maybe in combination with plaiting the wires should be done. Another option possibly arises through development of a metal detection in conjunction with an automatic deactivation (e.g. the holmium:YAG laser) in order to prevent the melting of a wire. A guided laser system in future should discriminate wire, stone and organic tissue.

## Conclusion

This was the first attempt at making stone baskets more resistant to a Holmium:YAG laser beam. Titanium oxide deposited by a sol–gel-process on a titanium-nickel alloy did not result in better resistance to laser injuries.

## Competing interests

The authors declare that they have no competing interests.

## Authors’ contributions

FH coated the wires. JC, FN and BL carried out the studies, JC drafted the manuscript. All authors read and approved the final manuscript.
